# The Kv2.1 K^+ ^channel targets to the axon initial segment of hippocampal and cortical neurons in culture and *in situ*

**DOI:** 10.1186/1471-2202-9-112

**Published:** 2008-11-13

**Authors:** Patrick D Sarmiere, Cecile M Weigle, Michael M Tamkun

**Affiliations:** 1 Program in Molecular, Cellular, and Developmental Neuroscience, Department of Biomedical Sciences & Department of Biochemistry and Molecular Biology, Colorado State University, Fort Collins, Colorado, USA; 2 Spinal Cord Society Research Center, 2401 Research Blvd. Suite 206, Fort Collins, Colorado, USA

## Abstract

**Background:**

The Kv2.1 delayed-rectifier K^+ ^channel regulates membrane excitability in hippocampal neurons where it targets to dynamic cell surface clusters on the soma and proximal dendrites. In the past, Kv2.1 has been assumed to be absent from the axon initial segment.

**Results:**

Transfected and endogenous Kv2.1 is now demonstrated to preferentially accumulate within the axon initial segment (AIS) over other neurite processes; 87% of 14 DIV hippocampal neurons show endogenous channel concentrated at the AIS relative to the soma and proximal dendrites. In contrast to the localization observed in pyramidal cells, GAD positive inhibitory neurons within the hippocampal cultures did not show AIS targeting. Photoactivable-GFP-Kv2.1-containing clusters at the AIS were stable, moving <1 *μ*m/hr with no channel turnover. Photobleach studies indicated individual channels within the cluster perimeter were highly mobile (FRAP *τ *= 10.4 ± 4.8 sec), supporting our model that Kv2.1 clusters are formed by the retention of mobile channels behind a diffusion-limiting perimeter. Demonstrating that the AIS targeting is not a tissue culture artifact, Kv2.1 was found in axon initial segments within both the adult rat hippocampal CA1, CA2, and CA3 layers and cortex.

**Conclusion:**

In summary, Kv2.1 is associated with the axon initial segment both *in vitro *and *in vivo *where it may modulate action potential frequency and back propagation. Since transfected Kv2.1 initially localizes to the AIS before appearing on the soma, it is likely multiple mechanisms regulate Kv2.1 trafficking to the cell surface.

## Background

Voltage-gated ion channels are often highly localized in electrically excitable cells such as nerve and muscle. As originally noted by Trimmer and colleagues [1], the Kv2.1 delayed rectifier is expressed primarily in the somatic region of hippocampal neurons where it is found in cell surface clusters that can co-localize with ryanodine receptors and SR-like subsurface cisterns [2,3]. Interestingly, these clusters also co-localize with cholinergic synapses in spinal motor neurons [4]. Kv2.1 represents the predominant delayed rectifier current in hippocampal neurons where its activity and localization are highly regulated [5,6]. Glutamate or carbachol treatments induce both Kv2.1 dephosphorylation and declustering [7-9]. Both treatments also result in a 20 mV hyperpolarizing shift in the activation curve for I_K_. Chemically-induced ischemia also induces declustering, dephosphorylation, and the hyperpolarizing shift in the activation midpoint [8,9]. Similar regulation is observed in Kv2.1 transfected HEK cells [9]. These data suggest a strong link between cluster formation, channel phosphorylation, and the voltage-dependence of activation. The increase in channel activity that is linked to declustering has been proposed to be a neuro-protective response to hypoxia/ischemic insult [10]. However, Kv2.1 trafficking to the cell surface is also implicated in cortical neuron apoptosis [11,12], emphasizing that the trafficking and regulation of Kv2.1 must be under tight physiological control.

While it is commonly assumed that ion channel localization must involve static tethering to scaffolding proteins that in turn are linked directly to the cytoskeleton, our recent studies indicate that the Kv2.1 surface clusters are formed when mobile Kv2.1 channels are corralled behind a cortical actin-based fence [13]. This sub-membrane fence is selective towards only the confined channels, with other membrane proteins being free to cross it. Thus, the Kv2.1-containing surface clusters represent a new mechanism for the stable localization of ion channel proteins to specific cell surface domains. Our previous studies also indicate that the surface clusters are specialized surface sites for the membrane insertion of Kv2.1 channels, functioning as intracellular trafficking vesicle targets [14]. During the course of our studies we often observed GFP-Kv2.1 clusters forming in a single proximal neurite of a transfected hippocampal neuron. While the expression of Kv2.1 within the axon initial segment (AIS) of cultured hippocampal neurons has previously been referred to as a tissue culture artifact [8], AIS localization was often the only cell surface expression observed in an individual cell. The study presented here was initiated by this apparent contradiction between the literature and our data obtained in hippocampal neurons transfected with GFP-Kv2.1.

We report here that both transfected and endogenous Kv2.1 often show a real preference for the AIS in cultured hippocampal neurons. The Kv2.1 clusters within the AIS are similar to those found on the cell body in that they consist of mobile channels trapped by a perimeter fence. However, perhaps due to the sub-membrane diffusion barriers in the AIS [15-17], the clusters themselves appear to be more confined than their cell body counterparts [14]. Kv2.1 concentration within the AIS also occurs in both cortical and hippocampal neurons of adult brain, confirming that AIS localization is not a tissue culture artifact. AIS-localized Kv2.1 is predicted to regulate both the frequency and back propagation of the axonal action potential.

## Results

### Exogenously expressed Kv2.1 targets to the axon initial segment in cultured hippocampal neurons before accumulating on the soma

**Figure 1:**
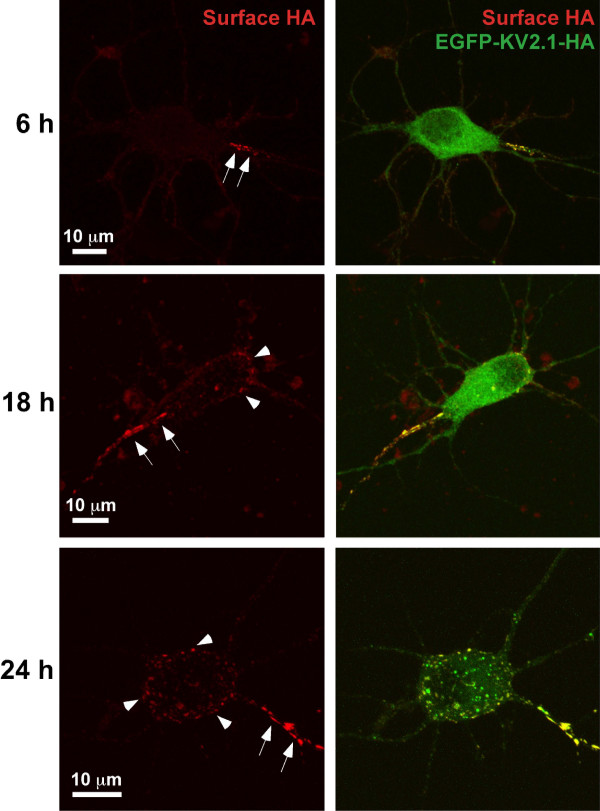
**Surface accumulation of transfected Kv2.1 in a single neurite**. Hippocampal neurons transfected with EGFP-Kv2.1-HA for 6, 18 and 24 h were labeled with Alexa 594-conjugated anti-HA monoclonal antibody 30 min prior to live cell imaging. Shown are representative maximum projection images comprised of multiple 0.3 *μ*m optical sections for total EGFP-Kv2.1-HA (green, GFP signal) and surface (red, Alexa 594 anti-HA antibody binding). Kv2.1 surface clusters were observed exclusively in single a proximal neurite as early as 6 hours after transfection (arrows). At 18 and 24 hours post-transfection, the appearance of surface somato-dendritic clustering became apparent (arrowheads).

Our initial imaging experiments involved expressing GFP and HA epitope-tagged Kv2.1 (EGFP-Kv2.1-HA) in rat hippocampal neurons grown in culture for 7–9 days. The extracellular HA epitope allowed detection of surface channel via the binding of an anti-HA Alexa-594-conjugated antibody to living cells as previously described [14]. In contrast to the expected soma and proximal neurite expression [7,18], 85% of neurons expressing EGFP-Kv2.1-HA exhibited surface accumulation at only one of the proximal neurites at 6 h post transfection (see arrows in Fig. 1, 6 h). This targeting of Kv2.1 to a single proximal neurite was observed with little or no clustering on the cell body. In neurons expressing Kv2.1 for 18 h, surface clusters within the soma (arrowheads) became more apparent relative to that observed at 6 h postransfection (Fig. 1, 18 h). By 24 h after transfection, the classic distribution of Kv2.1 into compact surface clusters was observed on the cell body along with expression in proximal neurites. However, Kv2.1 surface density remained the greatest in a single proximal neurite (Fig. 1, 24 h). Note that the majority of the cell body GFP fluorescence at the 6 h and 18 h time points most likely represents ER-localized channel. These representative data, showing that expressed Kv2.1 is targeted to a single proximal neurite prior to clustering at other surface somato-dendritic sites, suggest that Kv2.1 may have a preference for the axon initial segment (AIS) and may be targeted to this region via a mechanism distinct from the one responsible for delivery to somatic sites.

**Figure 2:**
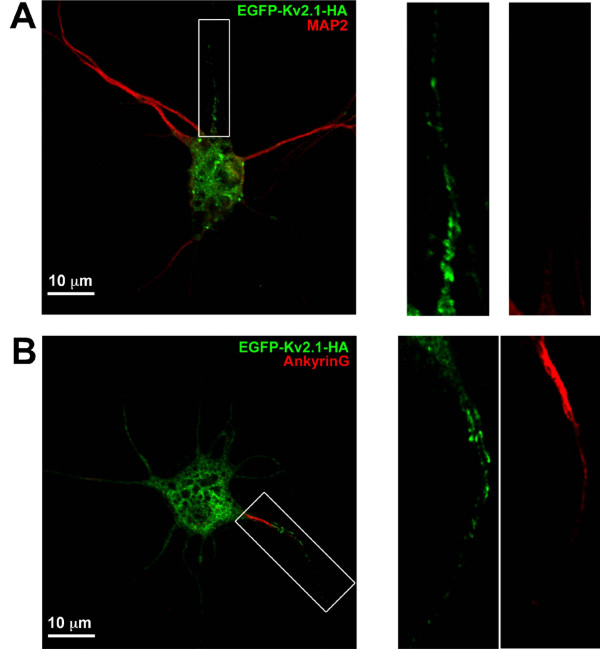
**The single proximal neurite containing Kv2.1 corresponds to the axon initial segment**. Hippocampal neurons grown for 10 days were transfected with EGFP-Kv2.1-HA and fixed 18 h later. Cultures were then labeled with either anti-MAP2 or anti-AnkG followed by Alexa 594-conjugated secondary antibodies. Shown in panel A is an example of Kv2.1 clustering in the proximal axon as indicated by the diminished MAP2 staining. Panel B illustrates intense AnkG staining in the Kv2.1 positive process. The right hand panels contain enlarged images of the neurites boxed in Panels A and B. Note that the diffuse GFP signal in the neurites represents intracellular GFP-Kv2.1.

A defining characteristic of the AIS in hippocampal neurons is the enrichment of ankyrinG (AnkG) in the first 20–40 *μ*m of the unmyelinated axon [19,20]. In addition, MAP2 immuno-reactivity is greatly reduced within the axon but is maintained within dendrites and the cell body [7,18]. Thus, MAP2 and AnkG expression were used to define the AIS in our culture system. Neurons transfected with EGFP-Kv2.1-HA were fixed approximately 18 h after transfection and immuno-stained for MAP2 or AnkG as illustrated in Fig. 2. The clustering of Kv2.1 in the proximal segment of a neurite with reduced MAP2 labeling suggests this neurite is likely to develop into the axon and therefore the proximal AIS (Fig. 2A). However, a stronger indication that the region of Kv2.1 accumulation is indeed the AIS is the AnkG staining shown in Fig. 2B. Consistent with AIS formation in hippocampal cultures, Nav1.2 co-localized with AnkG and was enriched in areas devoid of MAP2 staining in parallel cultures (data not shown).

### Endogenous Kv2.1 also preferentially localizes to the AIS

**Figure 3:**
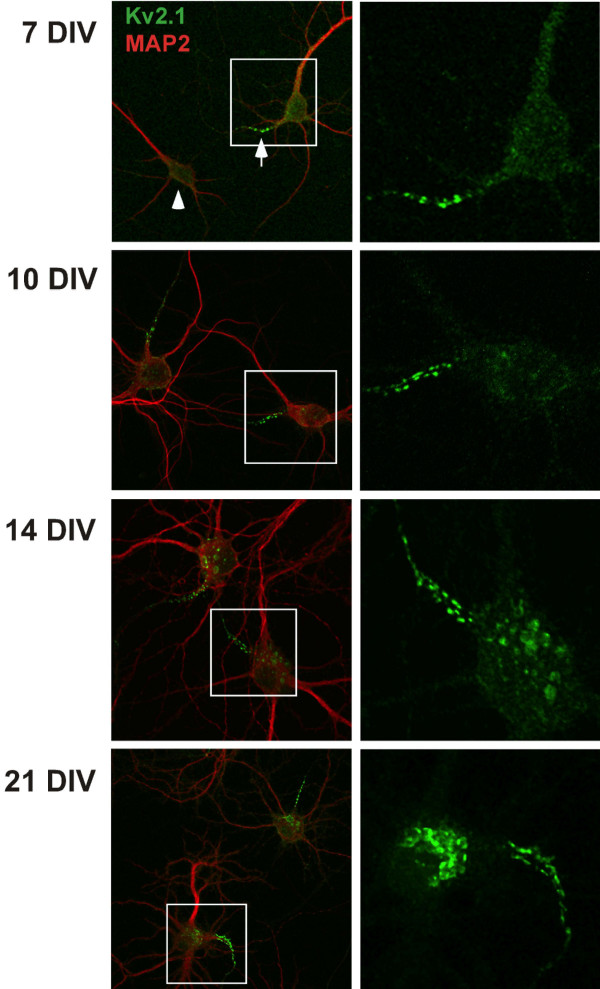
**Endogenous Kv2.1 preferentially accumulates in a MAP2-negative neurite**. Hippocampal cultures grown for 7, 10, 14 and 21 days were fixed and immunostained for endogenous Kv2.1 (green) and the dendritic marker MAP2 (red). At 7 DIV, the strongest labeling for Kv2.1 in these cultures appeared in the proximal portion of a MAP2-negative process (arrow) although many neurons exhibited faint clusters evenly dispersed over the cell body and proximal neurites (arrowhead). Shown in the right hand panels are maximum projection images corresponding to Kv2.1 staining from the boxed regions. In the majority of neurons, the neurite with intense Kv2.1 labeling corresponded to the process with the least intense MAP2 staining, indicating the preferential accumulation of Kv2.1 at the proximal axon.

Exogenous expression can lead to artifactual localization. To confirm that Kv2.1 is indeed a component of the AIS in hippocampal cultures, non-transfected cells were immuno-stained for endogenous Kv2.1 and MAP2. Kv2.1 localization in a single proximal neurite with diminished MAP2 staining, i.e. the AIS (marked by the arrow in Fig. 3), was observed in 35% of 7 DIV neurons and 63% of 10 DIV neurons (200 neurons examined in each of three experiments). The remaining cells showed no AIS localization and a clustered distribution primarily on the cell soma (arrowhead, Fig. 3) as previously reported [2]. Interestingly, as shown in Fig. 3, while all cells showed some Kv2.1 localization to the soma, the most intense staining in DIV 7 and 10 cultures was within the proximal axon. As the cells matured, i.e. in 14 and 21 DIV cultures, the cluster intensity within the cell soma became more equivalent to that in the AIS. We observed Kv2.1 expression on MAP2-positive neurites in only 10% of the 7 DIV neurons and 2% of the day 10 and 14 cells. Consistent with reports of multiple axon formation in this culture paradigm [21], 5 and 25% of the 7 and 14 DIV cells, respectively, showed Kv2.1 accumulation in two AIS domains. By the 14 DIV time point, approximately 87% of the cells showed dense Kv2.1 expression in one or two AIS domains along with expression over the soma. These expression patterns, and the percentage of cells expressing each pattern, are illustrated in Additional file 1. To further establish that the endogenous Kv2.1 accumulates at the AIS, we used antibodies directed against AnkG in conjunction with endogenous Kv2.1 immuno-detection. Consistent with AIS localization of Kv2.1, the sites of clustering within a proximal neurite corresponded with sites of AnkG enrichment (Additional file 2).

### Heterogeneity within the culture system

**Figure 4:**
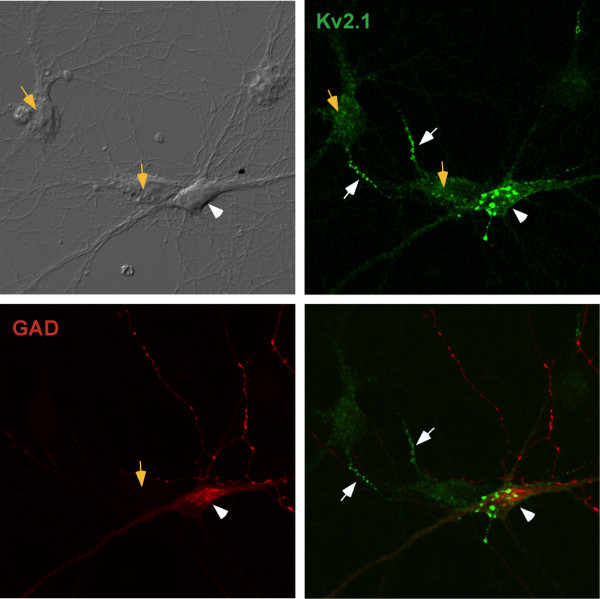
**GAD-positive cells do not exhibit Kv2.1 expression in the AIS**. DIV14 hippocampal neurons were fixed and stained for endogenous Kv2.1 and glutamic acid decarboxylase (GAD) to identify inhibitory neurons. The orange arrows indicate the GAD-negative neurons while the white arrowhead points to a GAD-positive cell. The white arrows indicate the AIS-localized Kv2.1 associated with the GAD-negative neurons. These results are consistent with Kv2.1 localization to the AIS only in excitatory, GAD-negative pyramidal neurons.

We did observe variability in the endogenous Kv2.1 expression pattern, for 12% of 14 DIV neurons showed no AIS localization. This heterogeneity is not surprising given that hippocampal cultures are heterotypic. Under culture conditions similar to ours, previous reports have placed the amount of pyramidal neurons between 80–90% whereas glutamic acid decarboxylase (GAD)-positive inhibitory interneurons are present at approximately 10–15% [2]. Hippocampal cultures were stained with an anti-GAD6 antibody in conjunction with anti-Kv2.1 staining to characterize the distribution of Kv2.1 in interneurons as compared to pyramidal neurons. As shown in Fig. 4, the GAD-positive neurons (white arrowhead) did not exhibit AIS localization of Kv2.1 while the GAD-negative neurons did (orange arrows). GAD-positive neurons did display large Kv2.1 clusters mainly within the cell body as previously described [2]. Together, these data suggest Kv2.1 preferentially targets to the proximal region of the pyramidal neuron axons (white arrows). Consistent with previous reports, 10% of the neurons in our culture were GAD-positive [2].

### AIS localized Kv2.1 clusters are stable structures containing mobile, but diffusion limited, channels

**Figure 5:**
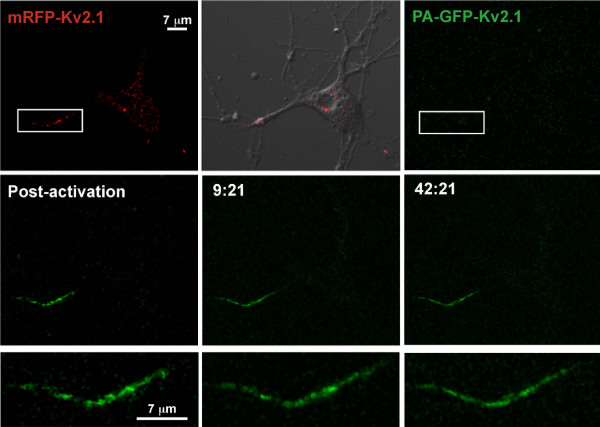
**Kv2.1 clusters within the AIS are stable**. Shown is a hippocampal neuron co-transfected with a 1:1 ratio of mRFP-Kv2.1 and PAGFP-Kv2.1-HA plasmid DNA. The proximal region of the single neurite expressing Kv2.1 (rectangle) was exposed to 405 nm laser light to photo-activate the PA-GFP. The top row illustrates the mRFP-Kv2.1 signal (red), the mRFP signal overlaid onto the corresponding DIC image and image acquisition under GFP optics. The middle row illustrates the PA-GFP signal immediately after photoactivation and at two representative time points (min:sec). The bottom row contains enlargements of the activated PA-GFP following photoactivation. Only small changes in cluster size and position occurred over the imaging period. There was no significant decrease in total AIS GFP fluorescence during the 42 minutes of acquisition, suggesting stable retention of Kv2.1 within the proximal axon. The imaging interval was 2 min.

Our previous work with the Kv2.1 clusters on the soma shows that while these structures are stable, they are also highly dynamic, often fusing and fragmenting over several min (see Movie S7 from [14]). To address the long term stability of the AIS localized Kv2.1 clusters, we utilized a photoactivatable form of GFP (PAGFP) fused to the N-terminus of Kv2.1 [22]. We co-transfected 7 DIV hippocampal neurons with equal amounts of mRFP-Kv2.1 and PAGFP-Kv2.1-HA and performed live cell image acquisition the following day. Taking advantage of the tetrameric nature of Kv channels, the mRFP-Kv2.1 construct was included solely to localize the AIS targeted channel for photoactivation. The AIS localized mRFP/PAGFP-Kv2.1 was selected for photoactivation under RFP optics (rectangle, Fig. 5) and successive imaging was performed every two minutes thereafter. As shown in Fig. 5, the photo-activated Kv2.1 within the AIS remained within the activation area and did not significantly re-distribute over the 42 min imaging period. There was no decrease in total AIS associated GFP fluorescence over the imaging period (8 × 10^5 ^verses 9.2 × 10^5 ^arbitrary units for the Fig. 5 example). These results imply that the Kv2.1 AIS clusters show little lateral diffusion within the membrane and that the channels contained within the AIS have residence times that will be measured in hours as opposed to minutes.

**Figure 6:**
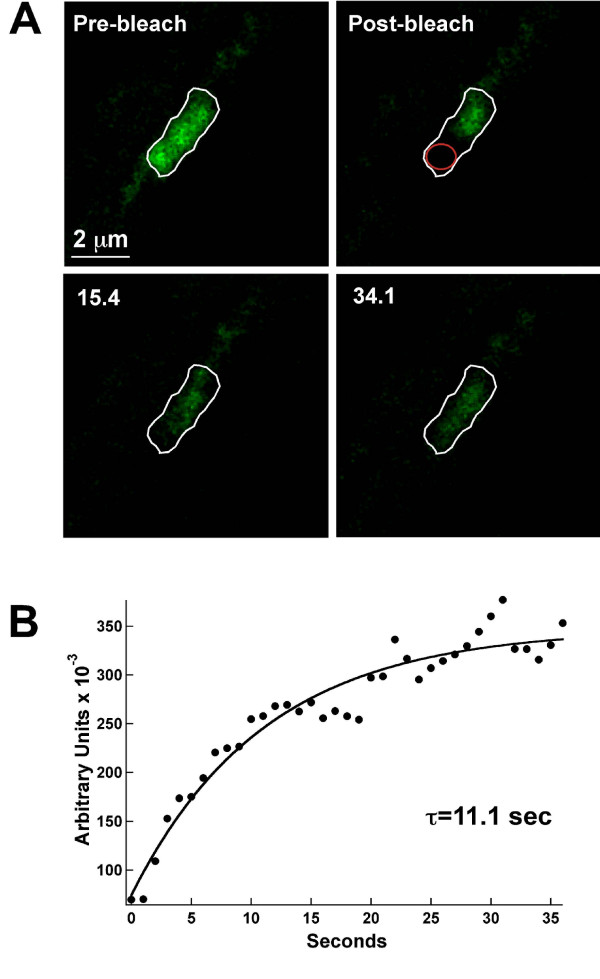
**FRAP analysis indicates Kv2.1 channels are mobile within the AIS cluster perimeter**. Panel A shows FRAP of an AIS cluster. One half of an AIS cluster (white outline) was photo-bleached and FRAP then monitored every 1.1 sec. By 15 sec into the recovery, GFP fluorescence was readily observed diffusing from the unbleached half into the bleached region. Panel B shows the FRAP time course. FRAP was quantitated within a small region of interest drawn in the bleached region (red circle). The solid line represents a single exponential fit to the data; *τ *= 11.1 sec for the cluster illustrated. The mean FRAP time constant for AIS clusters was 10.4 ± 4.8 sec, n = 7.

Our previous studies demonstrated that the Kv2.1 surface clusters on the hippocampal cell body are formed not by the tethering of individual channels to sub-membrane scaffolding proteins but rather by the trapping of mobile channels behind a cytoskeletal-based corral [13,14]. Given the stable nature of the AIS clusters illustrated in Fig. 5, we next sought to determine whether a similar localization mechanism existed within the AIS. As in our previous work [14], we used FRAP within the cluster to address Kv2.1 mobility within the cluster perimeter. As illustrated in Fig. 6, when one half of an AIS cluster was photo-bleached, GFP fluorescence recovery occurred rapidly with a time constant of 11.1 sec in this representative example. Overall, the mean time constant was 10.4 ± 4.8 sec, n = 7. These time constants are nearly identical to the 11.5 ± 6.1 sec value observed within clusters on the neuronal cell body [14]. This recovery, along with the corresponding decrease in fluorescence in the unbleached cluster half, is indicative of channel mobility within the cluster perimeter and is most consistent with mobile channels being restrained behind a perimeter fence in the AIS just as they are on the cell body.

### Kv2.1 localizes to hippocampal and cortical AIS domains in the intact rat brain

**Figure 7:**
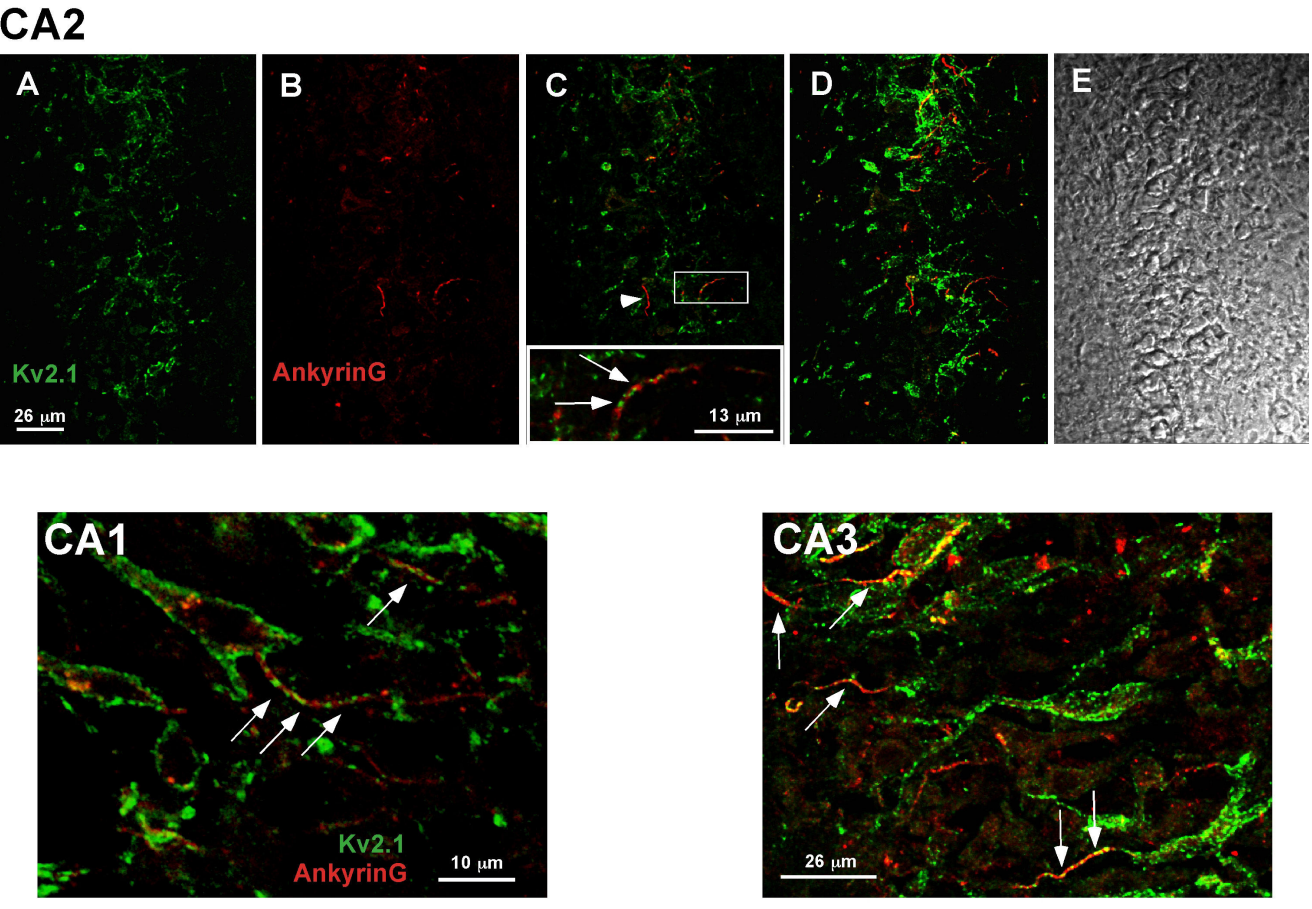
**Localization of Kv2.1 clusters to the AIS of hippocampal neurons *in situ***. Postnatal day 21 rat brains were formaldehyde-fixed, cryosectioned and immuno-stained with a polyclonal antibody against Kv2.1 and a monoclonal antibody against ankyrin G as described in Methods. The anti-Kv2.1 antibody was detected with Alexa 488-conjugated goat anti-rabbit secondary antibody (green) while the anti-AnkG monoclonal antibody was detected with Alexa 594-conjugated goat anti-mouse secondary (red). The top row of panels (A-E) illustrates the localization observed in the CA2 hippocampal layer. Single optical sections of either Kv2.1 or ankyrinG immuno-staining are shown in panels A and B, respectively. Panel C shows the overlay of these two images with the arrowhead pointing to an AIS domain with little or no Kv2.1 immuno-reactivity. However, as the insert containing a magnification of the boxed region indicates, Kv2.1 was most often found in the ankyrinG-positive AIS. Panel D shows a maximum projection image (Z-stack compression) and panel E the corresponding DIC image of the same field. The CA1 image represents a single optical section within the CA1 layer while the CA3 image is a maximum projection image of this hippocampal region. The arrows denote the expression of Kv2.1 within the AnkyrinG positive AIS domains.

The data presented thus far indicate that both the endogenous and exogenously expressed Kv2.1 accumulate at the AIS in hippocampal cultures, often preferring this region to other cell surface sites early in expression. To demonstrate that the robust AIS localization observed in our culture system is physiologically relevant, we undertook immuno-localization studies in rat brain. While Kv2.1 immuno-localization within the rat brain had been previously undertaken by several investigators [3,8], including ourselves (unpublished results), there was no previous suggestion that Kv2.1 exists within the AIS, probably due to the difficulty in identifying the relatively narrow AIS domains within the dense cell body layers of the hippocampus. However, as illustrated in Fig. 7, the use of AnkG staining to identify the AIS (arrows) greatly simplifies matters. Within the hippocampal CA2 layer, Kv2.1 clusters localized over the neuronal cell body as previously described [8] (Fig. 7, panels A-D). However, a linear array of Kv2.1 clusters was also observed within the boundaries of the AnkG positive AIS (arrows), up to 20 *μ*m from the cell body (Fig. 7, panel C inset). Consistent with the localization observed in our cultures of hippocampal neurons, not all the ankyrinG-defined AIS domains contained Kv2.1 clusters (arrowhead, panel C). AIS localization was also observed within the CA1 layer (Fig. 7, panel CA1) and CA3 (Fig. 7, panel CA3). Surprisingly, Kv2.1 immuno-localization was not detected in hippocampal sections prior to postnatal day 21. While channel might be diffusely expressed at these earlier developmental time points, the clustered localization typical of adult tissue was never observed. In adult tissue, the percentage of samples showing Kv2.1 positive AIS domains in the CA1, CA2, and CA3 hippocampal layers were 85 (n = 13), 64 (n = 11), and 100% (n = 17), respectively.

**Figure 8:**
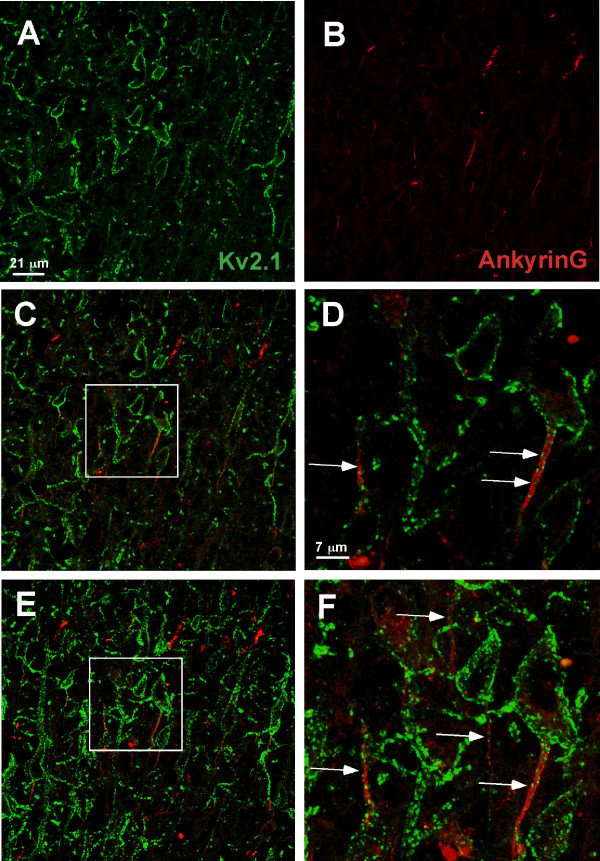
**Localization of Kv2.1 clusters to the AIS of layer IV cortical neurons *in situ***. Rat brain formaldehyde-fixed cyrosections obtained from post natal day 21 animals were immuno-stained with polyclonal antibody against Kv2.1 and monoclonal antibody against ankyrin G. The anti-Kv2.1 antibody was detected with Alexa 488-conjugated goat anti-rabbit secondary antibody (green) while the anti-AnkG monoclonal antibody was detected with Alexa 594-conjugated goat anti-mouse secondary antibody (red). Single optical sections of either Kv2.1 or ankyrinG immuno-staining are shown in panels A and B. Panel C shows the overlay of these two images and panel D contains a magnification of the boxed region in C. Panels E and F show a maximum projection image (Z-stack compression) corresponding to the areas shown in panels C and D. The arrows denote the expression of Kv2.1 within AnkG-positive AIS domains (arrows).

As shown in Additional file 3, we also observed AIS localization in cultured cortical neurons. Thus, Kv2.1 targeting to the AIS in cortical brain regions was examined also. Fig. 8 illustrates the localization of Kv2.1 to the neuron cell body and AIS *in situ *in cortical layer IV. In contrast to that observed in the hippocampus, localization of Kv2.1 clusters to the cell body and AIS in cortical sections was observed at early developmental times points, even occasionally at post natal day 2.

## Discussion

Any Kv2.1 localization to the AIS in cultured hippocampal neurons has been proposed to be a simple tissue culture artifact [8]. However, the data presented above indicate that both transfected and endogenous Kv2.1 often preferentially target to the AIS prior to accumulating at somatic cluster sites. Most importantly, we routinely detected AIS localization of Kv2.1 in the hippocampus and cortex of adult rats, suggesting a role for Kv2.1 in regulating axon excitability *in vivo*. The AIS-localized Kv2.1 channels reside within clusters similar to those found on the soma in that mobile channels are corralled within a perimeter fence [14]. However, the clusters themselves appear to be more stable within the AIS as compared to their cell body counterparts, consistent with ideas that cytoskeletal structures restrict the diffusion of membrane components within the AIS [16,17].

Our results indicate that transfected Kv2.1 accumulates at the AIS of hippocampal neurons prior to other cell surface sites (Fig. 1). Endogenous channel also concentrates at the AIS in 7 and 10 DIV neurons (Fig. 3). Given that the Kv2.1 surface clusters are sites where trafficking vesicles deliver channel to the cell surface [14], it is possible that vesicles containing newly synthesized Kv2.1 are delivered first to the AIS. A fraction of the AIS localized channel could then be internalized and redistributed over the soma. Another possibility is that Kv2.1 is inserted into the soma membrane and then diffuses into the AIS clusters where it becomes trapped. Such a diffusion-trap mechanism has been proposed for voltage-gated Na^+ ^channels that are AIS localized [23]. Alternatively, perhaps delivery occurs equally at the AIS and soma sites but since the AIS is the only neurite receiving the nascent channel, and the newly inserted channel is diluted over all the soma sites, we are left with only the appearance of selective AIS insertion. Future experiments will distinguish between these possibilities.

More than 87% of 14 DIV neurons demonstrate endogenous Kv2.1 concentration at the AIS. Why this localization has not been observed by other investigators is unclear. Perhaps subtle differences in tissue culture conditions could account for the discrepancy. Previous Kv2.1 localizations in adult brain, as performed by other investigators [3,8] and ourselves (unpublished results), also failed to detect Kv2.1 expression in the AIS. It is possible that the AIS localization was missed because without simultaneous ankyrinG staining and high resolution confocal imaging, it is difficult to identify the AIS structure within the dense layers of neuronal cell bodies in the hippocampus. The finding that not all hippocampal neurons show Kv2.1 localization in the AIS agrees with the Fig. 4 data showing a lack of AIS localization in GAD-positive inhibitory neurons.

## Conclusion

While a high concentration of sodium channels at the AIS ensures axonal action potential generation [24], it is not clear what role Kv2.1 plays in this specialized cell surface domain. Kv2.1 has slow activation kinetics and is therefore unlikely to contribute significantly to repolarization during a single action potential. However, Kv2.1 could regulate axonal excitability following trains of action potentials, for studies by McBain and co-workers indicate that reduction of Kv2.1 in pyramidal neurons results in a broadening of the action potential waveform [5]. Recently, the Kv2.2 channel, which cannot form heterotetrameric complexes with Kv2.1, has been localized to the AIS of neurons in the medial nucleus of the trapezoid body [25]. Kv2.2, which is functionally similar to Kv2.1, is proposed to enhance the recovery of AIS sodium channels from the inactive state by hyperpolarizing the membrane potential during repetitive action potential firing [25]. Kv2.1 could be performing a similar function. In addition, concentration of Kv2.1 at the AIS could regulate the back-propagation of depolarization into the soma. Given that multiple phosphorylation sites within the Kv2.1 C-terminus influence the voltage-dependence of activation [26], the phosphorylation of AIS-localized Kv2.1 provides a mechanism to regulate axonal excitability.

## Methods

### Primary hippocampal cell cultures

Neurons from embryonic day 18 rat pups were cultured as previously described [27]. Briefly, neurons from cryo-preserved E18 rat hippocampal dissociations were plated at a density of ~15,000 – 30,000 cells/cm2 on poly-D-lysine coated glass-bottom dishes (MatTek) in glial-cell conditioned neurobasal medium (GCM) containing B27 supplement (Invitrogen) [28]. Every 3–4 days after plating, one-half of the culture medium was replaced with GCM. Neurons plated on 35 mm glass bottom dishes and cultured for 7–10 days were transfected with 2.0 *μ*l of Lipofectamine 2000 and 0.75 *μ*g of Kv2.1-expressing plasmid DNA in 100 *μ*l OptiMem (Invitrogen). Two-hours after transfection, the culture medium was replaced with fresh Neurobasal/B27. The expression plasmid constructs containing fluorescent protein and epitope-tagged Kv2.1 have been previously described [13,14,22,29]. In brief, EGFP was placed onto the Kv2.1 N-terminus and two HA epitopes were inserted in the extracellular loop between S1 and S2 transmembrane domains.

### Live cell imaging

All image acquisition was performed using an Olympus Fluoview 1000 confocal microscope system as previously described [13,14]. For live cell acquisition, the objective and microscope stage were maintained at 37°C. Prior to imaging, media was replaced with pre-warmed Hepes-buffered (25 mM) Neurobasal medium containing B-27 supplement (Invitrogen). GFP was excited using the 488 nm line of an Ar laser set at 0.1 – 0.5% transmission and emission collected using the variable bandpass filter set at 500–550 nm. A 60×, 1.4 NA oil immersion objective was used for imaging and the pinhole diameter set for 1 Airy Unit. For each image, the detector voltage was adjusted as necessary to utilize the full 12-bit linear range. For the imaging of individual Kv2.1-containing clusters, an optical zoom of 8–10 × was often used. Images were acquired every 0.5 – 120 seconds as indicated at either 512 × 512 or 1024 × 1024 resolution. Cells were imaged for less than one hour on the microscope stage.

For FRAP analysis of GFP-Kv2.1 within the cluster perimeter, a circular region of interest (ROI) containing approximately half of the cluster area was photobleached using the SIM scanner of the Olympus FV1000 in tornado scan mode with a 405 nm diode laser at 12–20% transmission for 0.5–1 seconds as previously described [13,14]. The SIM scanner was synchronized with the main scanner during bleach and acquisition. Following bleach, imaging was performed by raster scanning with the 488 nm line of a 40 mW Ar laser at 0.2–0.5% transmission. The variable bandpass filter was set to detect 505 – 530 nm emission and images were acquired every 0.5–1.1 seconds at 512 × 512 resolution. Fluorescence intensity, in arbitrary units, within the bleach ROI was fit according to Eq. 1 as previously described [13,22].

(1) $$f(x)=\sum_{i=1}^{n}A_{i}(1-e^{\left(-t/\tau_{i}\right)})$$

where A_i _is the amplitude of each component, *t *is time and *τ*_i _is the time constant of each component.

### Immunofluorescence

Detection of surface GFP-Kv2.1-HA was performed by incubating live cells with a 1:2000 dilution of anti-HA Alexa-594 conjugated monoclonal antibody (Molecular Probes) in Hepes-buffered Neurobasal/B27 for a minimum of 30 min at 37°C. Only live cells were used for surface channel detection since fixation permits labeling of internal, non-surface channel (data not shown). For endogenous Kv2.1, ankyrinG and MAP2 immuno-staining, neurons were fixed in 4% formaldehyde, 4% sucrose in PBS for 15 min, incubated in 0.5% CHAPS, blocked in 5% non-fat milk and 1% goat serum in PBS, and labeled with the indicated antibody diluted in 1% BSA in PBS. Antibody and dilutions were as follows: affinity-purified rabbit anti-Kv2.1 polyclonal antibody (Upstate Biotechnology, 1:200 dilution, raised against amino acids 837–853); mouse monoclonal anti-MAP-2 (Sigma, 1:2000); mouse monoclonal anti-AnkyrinG (Zymed, 1:100, raised against the spectrin binding domain); and mouse monoclonal anti-*α*-glutamic acid decarboxylase (Developmental Studies Hybidoma Bank, 1:50). Goat anti-mouse and goat anti-rabbit secondary antibodies conjugated to Alexa 488 or 594 (Molecular Probes) were diluted 1:1000 in 1% BSA/PBS.

Hippocampal slices were prepared from post natal day 21 and adult rats. In accordance with university guidelines, the animals were anesthetized with ketamine and decapitated. After decapitation, the brains were removed and fixed with 4% paraformaldehyde (PFA) for 4 hours at 4°C, replacing the PFA solution after 2 hours. The tissue was cryoprotected in 30% sucrose for 2 days at 4°C. Following cryoprotection and freezing, sagittal sections (16 *μ*m) were prepared on a cryostat microtome. In order to diminish non-specific binding, the tissue sections were incubated overnight in 1 × PBS/5% dry milk/10% goat serum at 4°C. The detection of endogenous Kv2.1 and ankyrinG was performed by incubating the tissue sections with affinity-purified anti-Kv2.1 polyclonal (1:500, Upstate Biotechnology) and anti-Ankyrin G monoclonal (1:500, Zymed) antibodies diluted in 1 × PBS/10% goat serum/0.5% CHAPS overnight at 4°C. Following primary antibody incubation, brain slices were washed three times with 1 × PBS/0.5% CHAPS. The tissue slices were incubated with Alexa 488-conjugated goat anti-rabbit secondary (1:500, Invitrogen) and Alexa 594-conjugated goat anti-mouse secondary (1:500, Molecular Probes) for 1 hour at room temperature. Following secondary antibody incubation, the brain slices were washed three times with 1 × PBS/0.5% CHAPS and mounted using AquaPolymount (Polysciences).

### Specificity of antibody binding

The detection of MAP2, ankyrinG, GAD and Kv2.1 in cultured hippocampal neurons has been performed by multiple groups using either the same antibodies as used here or closely related ones [2,7,18,20,30-36]. Importantly, the immunolocalization patterns for endogenous Kv2.1 in the hippocampal cultures are identical to the patterns observed with GFP-Kv2.1-HA under GFP optics or with anti-HA immuno-staining. With respect to the anti-HA antibody specificity, cell surface staining was never observed in GFP-Kv2.1-HA free neurons.

An additional control is required for the Kv2.1 localization to the ankyrinG defined AIS in brain tissue sections, as this has not been reported previously. Since immunogen block controls are known to be misleading [37], we also performed immuno-localization of ankyrinG and Kv2.1 using a monoclonal antibody for Kv2.1 and a polyclonal antibody for ankyrinG. A mouse monoclonal against Kv2.1 (1:500 dilution, Upstate Biotechnology, raised against a GST fusion protein containing amino acids 509–853) was used in conjunction with a rabbit anti-ankyrinG polyclonal (1:500 dilution, Santa Cruz Biotechnology, raised against amino acids 4163–4377). While the immuno-staining was not as robust as that observed with the other antibody combination, the same AIS staining patterns for ankyrinG and Kv2.1 were observed as shown in Additional file 4. It is highly unlikely that these distinct anti-Kv2.1 antibodies both nonspecifically bind AIS structures, especially since GFP-Kv2.1-HA also targets to this region in transfected neurons.

### Image analysis

Offline image analysis was done using the Olympus FV1000 software (version 1.03) and Volocity 4.4 (Improvision, Lexington, MA). Data analysis and curve-fitting was performed with SigmaPlot 8 (Systat, Point Richmond, CA) or IgorPro 5.03 (Wavemetrics, Portland, OR). Images were filtered in Volocity using a 3 × 3 median filter. Compilation of images was performed using Adobe Photoshop and contrast and brightness adjustments were made. Both compressed (maximum projection) and single Z-section images are displayed as indicated.

## Abbreviations

AIS: Axon initial segement; AnkG: Ankyrin G; DIV: Days *in vitro*; GAD: glutamic acid decarboxylase.

## Authors' contributions

PDS and MMT designed the experimental procedures, prepared *in vitro *cultures, and performed imaging and analysis. CMW performed tissue sectioning and immunostaining. The article was written by PDS and MMT.

## Supplementary Material

Additional file 1 **Summary of the endogenous Kv2.1 localization patterns in cultured hippocampal neurons**. The bar graph of panel A illustrates the percentages of neurons in 7, 10, and 14 DIV cultures that showed the expression patterns illustrated in B. Neurons were formaldehyde-fixed and immuno-stained with polyclonal antibody against Kv2.1 and monoclonal antibody against MAP2. The anti-Kv2.1 antibody was detected with Alexa 488-conjugated goat anti-rabbit secondary antibody (green) while the anti-MAP2 monoclonal antibody was detected with Alexa 594-conjugated goat anti-mouse secondary antibody (red). The images represent a maximum projection image. The arrows denote the expression of Kv2.1 within a proximal neurite. Click here for file

Additional file 2 **Endogenous Kv2.1 localizes to the axon initial segment as defined by AnkyrinG enrichment**. Hippocampal neurons grown for 14 DIV were fixed and immuno-stained for the AIS marker AnkyrinG and endogenous Kv2.1. Shown are superimposed images of neurons stained for Kv2.1 (green) and AnkyrinG (red) from single basal z-sections. Individual fluorescent images, corresponding to boxed regions in the overlay, are shown to the right. Click here for file

Additional file 3 **Localization of endogenous Kv2.1 to the AIS of cultured cortical neurons**. Cortical neurons were isolated from E18 rat cortex by mechanical dissociation following treatment with 0.25% trypsin. Cells were grown on poly-lysine coated glass bottomed dishes in Neurobasal/B27/PenStrep for 10 days were formaldehyde-fixed and immuno-stained with polyclonal antibody against Kv2.1 and monoclonal antibody against MAP2. The anti-Kv2.1 antibody was detected with Alexa 488-conjugated goat anti-rabbit secondary antibody (green) while the anti-MAP2 monoclonal antibody was detected with Alexa 594 conjugated goat anti-mouse secondary antibody (red). The image represents a single optical section at the level of the substrate-attached neurites. The arrows denote the expression of Kv2.1 within a MAP2 negative neurite that is defined as the AIS. Click here for file

Additional file 4 **Localization of Kv2.1 to the AIS in the CA1 layer using a monoclonal anti-Kv2.1 antibody paired with a polyclonal anti-ankyrinG antibody**. Postnatal day 21 rat brains were formaldehyde-fixed, cryosectioned and immuno-stained with a monoclonal antibody against Kv2.1 and polyclonal clonal antibody against ankyrinG as described in Methods. The anti-Kv2.1 antibody was detected with Alexa 594-conjugated goat anti-mouse secondary antibody (red) while the anti-ankyrinG monoclonal antibody was detected with Alexa 488-conjugated goat anti-rabbit secondary (green). The image represents a single optical section. The arrows denote the expression of Kv2.1 within the AnkG positive AIS domain. Click here for file
