# Supplementary figures and images for: The Kv2.1 K+ channel targets to the axon initial segment of hippocampal and cortical neurons in culture and in situ
Source: BMC Neurosci. 2008 Nov 13;9:112. doi: 10.1186/1471-2202-9-112 (PMC2592246; doi:10.1186/1471-2202-9-112)

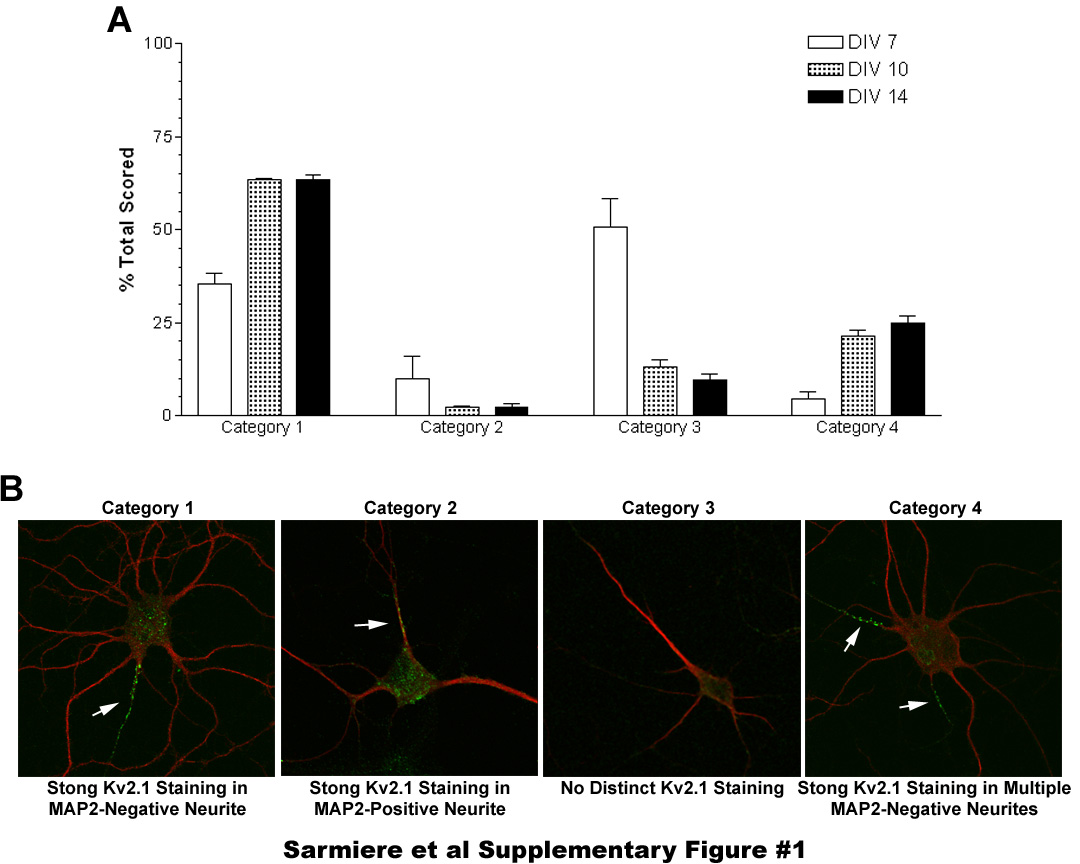

Supplement: Additional file 1 — Summary of the endogenous Kv2.1 localization patterns in cultured hippocampal neurons. The bar graph of panel A illustrates the percentages of neurons in 7, 10, and 14 DIV cultures that showed the expression patterns illustrated in B. Neurons were formaldehyde-fixed and immuno-stained with polyclonal antibody against Kv2.1 and monoclonal antibody against MAP2. The anti-Kv2.1 antibody was detected with Alexa 488-conjugated goat anti-rabbit secondary antibody (green) while the anti-MAP2 monoclonal antibody was detected with Alexa 594-conjugated goat anti-mouse secondary antibody (red). The images represent a maximum projection image. The arrows denote the expression of Kv2.1 within a proximal neurite. [file 1471-2202-9-112-S1.jpeg]

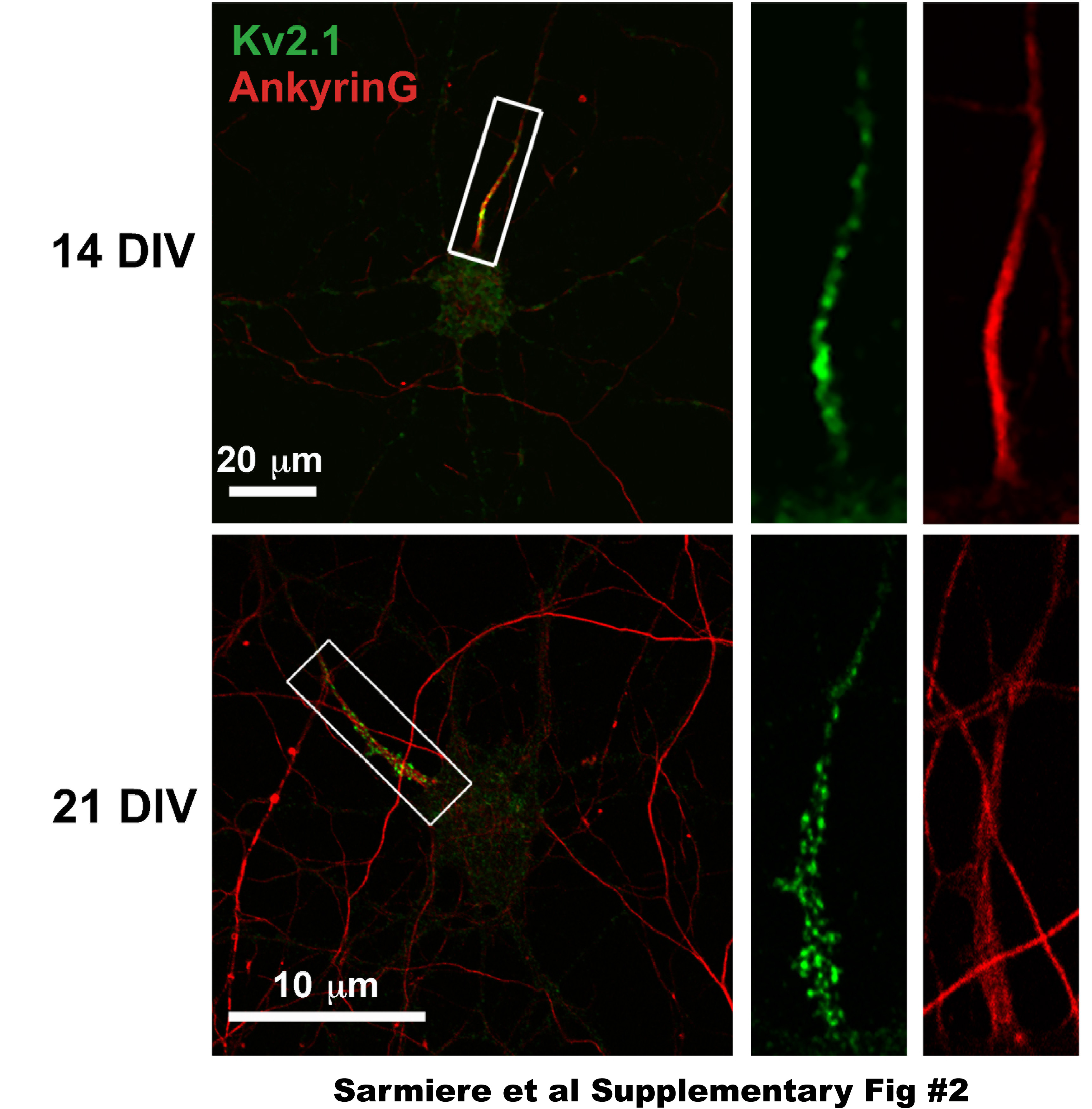

Supplement: Additional file 2 — Endogenous Kv2.1 localizes to the axon initial segment as defined by AnkyrinG enrichment. Hippocampal neurons grown for 14 DIV were fixed and immuno-stained for the AIS marker AnkyrinG and endogenous Kv2.1. Shown are superimposed images of neurons stained for Kv2.1 (green) and AnkyrinG (red) from single basal z-sections. Individual fluorescent images, corresponding to boxed regions in the overlay, are shown to the right. [file 1471-2202-9-112-S2.jpeg]

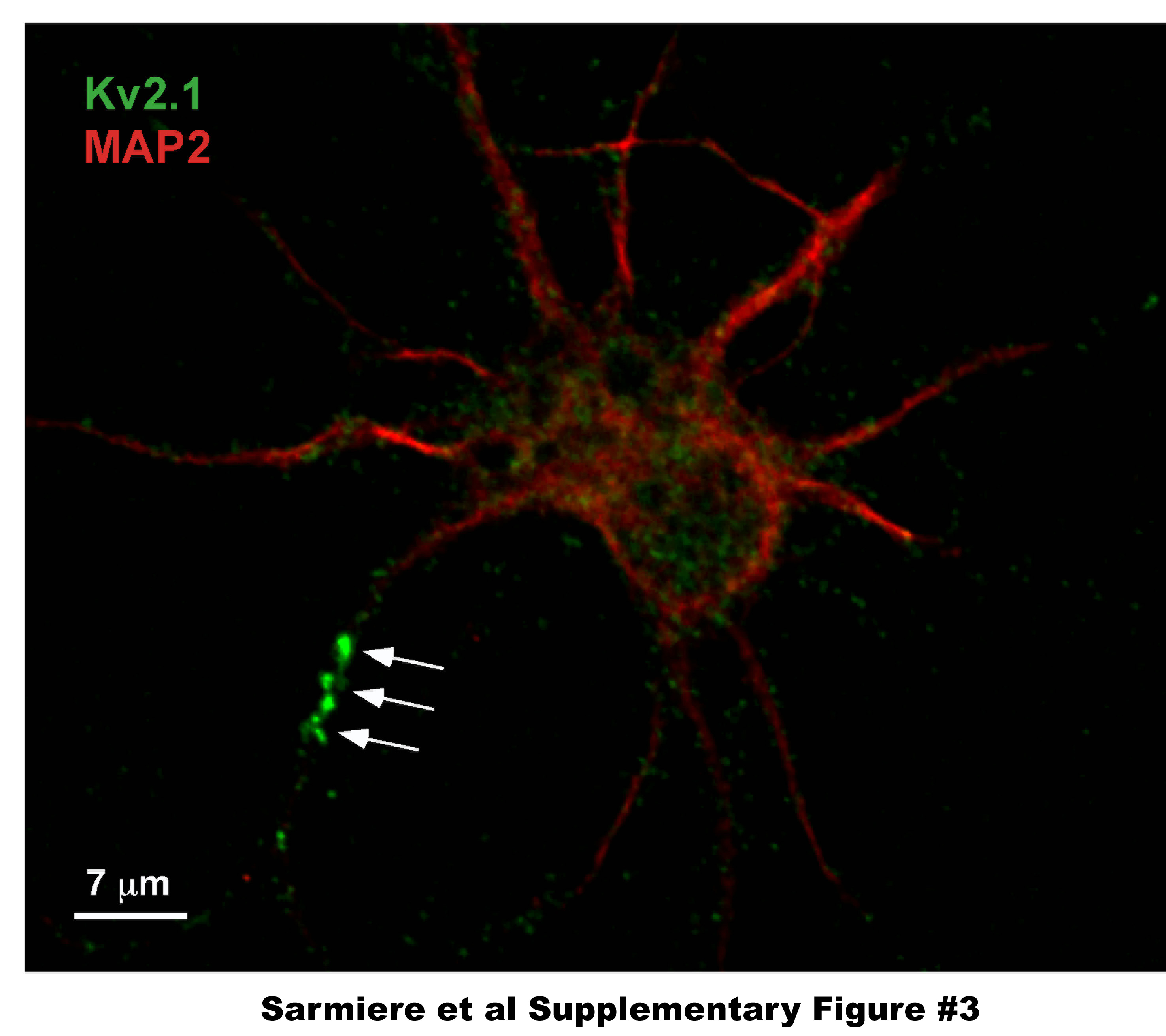

Supplement: Additional file 3 — Localization of endogenous Kv2.1 to the AIS of cultured cortical neurons. Cortical neurons were isolated from E18 rat cortex by mechanical dissociation following treatment with 0.25% trypsin. Cells were grown on poly-lysine coated glass bottomed dishes in Neurobasal/B27/PenStrep for 10 days were formaldehyde-fixed and immuno-stained with polyclonal antibody against Kv2.1 and monoclonal antibody against MAP2. The anti-Kv2.1 antibody was detected with Alexa 488-conjugated goat anti-rabbit secondary antibody (green) while the anti-MAP2 monoclonal antibody was detected with Alexa 594 conjugated goat anti-mouse secondary antibody (red). The image represents a single optical section at the level of the substrate-attached neurites. The arrows denote the expression of Kv2.1 within a MAP2 negative neurite that is defined as the AIS. [file 1471-2202-9-112-S3.jpeg]

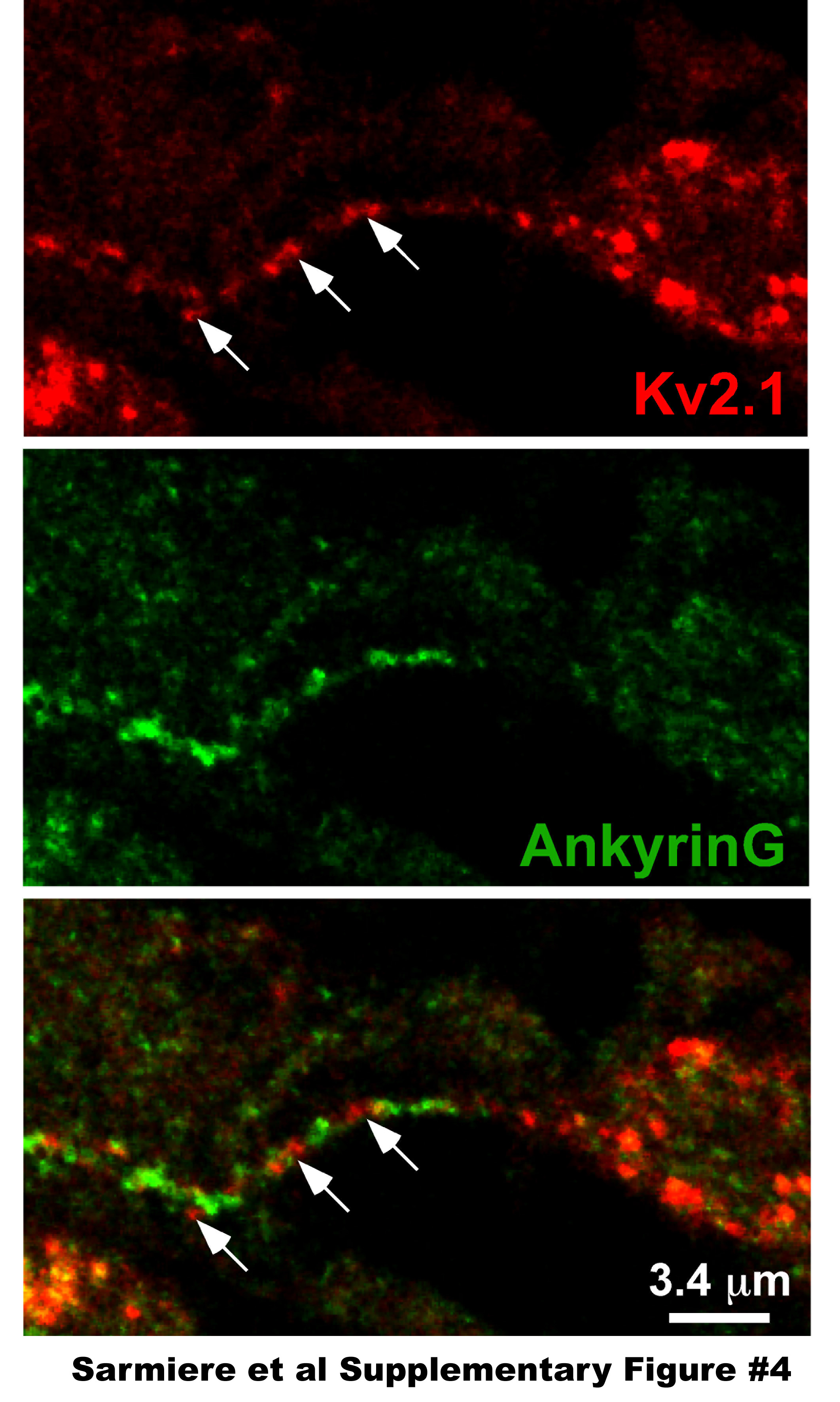

Supplement: Additional file 4 — Localization of Kv2.1 to the AIS in the CA1 layer using a monoclonal anti-Kv2.1 antibody paired with a polyclonal anti-ankyrinG antibody. Postnatal day 21 rat brains were formaldehyde-fixed, cryosectioned and immuno-stained with a monoclonal antibody against Kv2.1 and polyclonal clonal antibody against ankyrinG as described in Methods. The anti-Kv2.1 antibody was detected with Alexa 594-conjugated goat anti-mouse secondary antibody (red) while the anti-ankyrinG monoclonal antibody was detected with Alexa 488-conjugated goat anti-rabbit secondary (green). The image represents a single optical section. The arrows denote the expression of Kv2.1 within the AnkG positive AIS domain. [file 1471-2202-9-112-S4.jpeg]
